# An Emerging DTMUV Variant Remains Restricted in Mammals but Acquires Olfactory Route‐Mediated Neuroinvasion Following Limited Adaptive Mutations

**DOI:** 10.1155/tbed/4857491

**Published:** 2026-08-18

**Authors:** Qing Yang, Ting Zhou, Zhengzheng Li, Kang Yang, Chenyang Song, Qingqing Du, Yingqi Zhu, Guijun Wang

**Affiliations:** ^1^ College of Veterinary Medicine, Anhui Agricultural University, Hefei 230036, China, ahau.edu.cn; ^2^ Shanghai Veterinary Research Institute, Chinese Academy of Agricultural Sciences, Shanghai 200241, China, caas.cn

**Keywords:** Duck tembusu virus, mammalian adaptation, neuroinvasion, olfactory route, species barrier

## Abstract

Duck Tembusu virus (DTMUV), a mosquito‐borne flavivirus, has caused widespread outbreaks in poultry and has exhibited an expanding host range among avian species, accompanied by ongoing genetic variation and adaptation. As many mosquito‐borne flaviviruses can infect mammals and cross species barriers, it remains unclear whether recently emerged DTMUV variants have acquired similar potential. To address this, we assessed whether the circulating variant AQ‐19 can establish infection in mammals via peripheral routes. Despite its enhanced virulence in avian species, this variant failed to establish infection in mice following peripheral inoculation, indicating that a substantial barrier still restricts cross‐species transmission to mammals. We next investigated whether additional adaptive changes are required for DTMUV to overcome this barrier. Through serial intracranial passage in Institute of Cancer Research (ICR) mice, we generated a mouse‐adapted strain (F13) that exhibited markedly enhanced virulence and, notably, acquired the ability to invade the central nervous system (CNS) via intranasal (i.n.) inoculation, resulting in lethal infection. This neuroinvasive phenotype was strictly route‐dependent, as infection remained ineffective via intraperitoneal (i.p.) and subcutaneous (s.c.) routes. We found that the adapted virus invades the CNS via the olfactory route and disseminates broadly, exhibiting strong neurotropism and inducing severe neural damage. Functional disruption of the olfactory epithelium using ZnSO_4_ significantly delayed disease onset and reduced viral burden in the brain, supporting its role as a major route for nasal‐to‐brain transmission. Importantly, genomic analysis identified only four nonsynonymous amino acid substitutions in the adapted strain, suggesting that a limited number of mutations may be sufficient to confer enhanced neurovirulence and enable neuroinvasion in a mammalian host. Collectively, these findings demonstrate that the emerging DTMUV variant AQ‐19 remains restricted in mammals, whereas limited adaptive changes acquired during experimental mouse adaptation are associated with the acquisition of neuroinvasive capacity, providing new insights into the evolutionary potential of DTMUV for mammalian adaptation.

## 1. Introduction

Duck Tembusu virus (DTMUV), a mosquito‐borne flavivirus, has caused widespread outbreaks in poultry and poses a persistent threat to the avian industry [1–4]. Since its emergence, DTMUV has exhibited an expanding host range, extending beyond ducks to include geese [5, 6], chickens [7, 8], and other bird species [9]. Concurrently, accumulating evidence indicates that the virus is undergoing continuous genetic variation and adaptation, with recent circulating strains displaying increased virulence and enhanced neurotropic characteristics in avian hosts [10, 11]. These evolutionary trends suggest that DTMUV is actively adapting to new hosts and may be approaching a broader host spectrum.

Many mosquito‐borne flaviviruses, including Japanese encephalitis virus (JEV) [12], West Nile virus (WNV) [13], and Zika virus (ZIKV) [14], are capable of infecting mammals and frequently exhibit neurotropism, leading to central nervous system (CNS) infection and neurological disease [15, 16]. These viruses can cross species barriers and adapt to mammalian hosts, highlighting the intrinsic zoonotic potential within the flavivirus genus. In contrast, although DTMUV has been primarily associated with avian hosts, serological and molecular evidence from duck farm workers suggests potential human exposure [17]. Notably, recent evidence has reported the natural infection of a marine mammal (bottlenose dolphin) by DTMUV [18], indicating that the virus may possess a previously unrecognized capacity for cross‐species transmission.

Despite accumulating evidence suggesting potential cross‐species transmission, the ability of DTMUV to infect mammals remains poorly defined. Previous studies have shown that DTMUV can induce neurological symptoms and mortality in mice following intracranial inoculation, demonstrating substantial neurovirulence [19, 20]. Neurovirulence refers primarily to the ability of a virus to replicate and cause pathological damage after gaining access to the CNS, whereas neuroinvasion describes its capacity to overcome barriers outside the CNS and enter neural tissues from peripheral sites. However, infection via peripheral routes, such as intraperitoneal (i.p.) or subcutaneous (s.c.) administration, fails to establish productive infection, indicating a lack of effective neuroinvasive capacity in mammalian hosts [21, 22]. These findings reveal a critical discrepancy between neurovirulence and neuroinvasion and suggest the presence of a species barrier that restricts DTMUV transmission from avian hosts to mammals. However, the extent to which this barrier can be overcome, and the viral determinants required for this transition, remain unknown.

In this study, we sought to determine whether a recently circulating DTMUV variant with enhanced virulence in avian hosts has the potential to overcome species barriers and infect mammals, and to define the extent of viral adaptation required for this transition. Using the AQ‐19 strain as a representative variant, we first evaluated its ability to establish infection in mice via peripheral routes. We further investigated whether limited adaptive changes could enable DTMUV to acquire neuroinvasive capacity in a mammalian host. Through a mouse adaptation model, we demonstrate that minimal genetic changes are sufficient to confer neuroinvasion via the olfactory route, providing new insights into the evolutionary threshold required for cross‐species transmission of flaviviruses.

## 2. Materials and Methods

### 2.1. Cell and Virus

Baby Hamster Syrian Kidney cells (BHK‐21, ATCC, CCL‐10) were grown in Dulbecco’s modified Eagle Medium (DMEM, Gibco, USA) with 10% fetal bovine serum (FBS, Gibco, USA), 100 U/mL penicillin, and 100 mg/mL streptomycin (SenBeiJia Biological Technology, Nanjing, China). The BHK‐21 cells were incubated at 37°C in a CO_2_ incubator before use. The DTMUV AQ‐19 strain used in this study was isolated in our laboratory in 2019 (GenBank Accession: MT708901). The virus was propagated in BHK‐21 cells, and the viral titers were determined using the Reed and Muench method as the median tissue culture infective dose (TCID_50_)/0.1 mL. The titer of the stock DTMUV was 10^6^ TCID_50_/0.1 mL.

### 2.2. Ethical Considerations

We confirmed that all animal experiments were conducted following the Guide for the Care and Use of Laboratory Animals set by Anhui Agricultural University. The Institutional Animal Care and Use Committee of Anhui Agricultural University approved the protocols under protocol permit number AHAUB2022018.

### 2.3. Evaluation of the Neurovirulence and Neuroinvasive Capacity of Parental AQ‐19 in Mice

Three‐week‐old Institute of Cancer Research (ICR) mice were randomly assigned to three groups: a control group, an AQ‐19 intracerebral inoculation group, and an AQ‐19 intranasal (i.n.) inoculation group, with 10 mice per group. Mice in the intracerebral group were inoculated with 30 μL of AQ‐19 at a titer of 10^6^ TCID_50_/0.1 mL, corresponding to ~3 × 10^5^ TCID_50_ per mouse, under anesthesia. Mice in the i.n. group were inoculated without anesthesia with 100 μL of AQ‐19 at the same titer, corresponding to 1 × 10^6^ TCID_50_ per mouse. Control mice received an equivalent volume of serum‐free DMEM.

All mice were monitored daily for 14 days for clinical signs, body‐weight changes, and survival. Body weight was recorded for all surviving mice at each observation time point. Clinical observations included activity level, posture, coat condition, body condition, gait, and neurological abnormalities. Depression was defined as reduced spontaneous activity and responsiveness to external stimulation; hind limb paralysis was defined as partial or complete loss of voluntary movement in one or both hind limbs; and emaciation was defined as marked loss of body condition accompanied by prominent skeletal contours and sustained body‐weight reduction. Mice showing severe neurological dysfunction, inability to access food or water, or a moribund condition were humanely euthanized and recorded as deaths for survival analysis. Body‐weight change and survival were used as the principal quantitative indicators of disease severity, whereas the onset and progression of neurological signs, particularly hind limb paralysis, were recorded as key clinical indicators. Mice that survived until 14 days postinoculation (dpi) were humanely euthanized at the end of the experiment.

Brain tissues were collected from three mice per group for gross pathological examination, RT‐PCR detection, histopathological analysis, and immunohistochemistry. In mice that developed disease, samples were collected at death or when humane endpoints were reached. In mice that remained clinically normal, samples were collected at 14 dpi. The same tissue specimens were used for gross examination and were subsequently divided for virological and histopathological analyses.

### 2.4. Serial Intracranial Passage and Generation of Mouse‐Adapted DTMUV

To generate a mouse‐adapted DTMUV strain, 3‐week‐old ICR mice obtained from the Animal Experiment Center of Anhui Medical University (Hefei, China) were used. For each passage, 10 mice were intracerebrally inoculated with 30 μL of virus at a titer of 10^6^ TCID_50_/0.1 mL, corresponding to ~3 × 10^5^ TCID_50_ per mouse. Mice were monitored daily for clinical signs, including reduced activity, rough coat, emaciation, gait abnormalities, and neurological manifestations. Brain tissues were collected from animals that developed evident neurological signs, severe emaciation, or reached predefined humane endpoints, including the inability to access food or water or a moribund condition. No fixed interval was applied between passages. Each subsequent passage was initiated after affected mice from the preceding passage developed evident neurological signs or reached the predefined humane endpoints, followed by preparation, cell propagation, and titration of the recovered virus.

Brain tissues from five affected mice were pooled and homogenized in serum‐free Dulbecco’s modified Eagle medium (DMEM) at a ratio of 1 g of tissue to 9 mL of medium to prepare a 10% (w/v) homogenate. The homogenate was clarified by centrifugation at 12,000 rpm for 15 min at 4°C, and the resulting supernatant was passed through a 0.22‐μm filter. A 100‐μL aliquot of the filtered supernatant was inoculated onto BHK‐21 cells cultured in one well of a six‐well plate. When an evident cytopathic effect was observed, the infected cells and culture supernatant were subjected to one freeze–thaw cycle and centrifuged at 12,000 rpm for 5 min at 4°C. The clarified supernatant was collected as the corresponding passage virus. The infectious titer of each passage virus was determined using the TCID_50_ assay and adjusted to 10^6^ TCID_50_/0.1 mL before intracerebral inoculation of the next group of mice. This procedure was repeated for 13 consecutive passages, resulting in the generation of the mouse‐adapted F13 strain.

### 2.5. Comparative Neurovirulence of AQ‐19 and Serially Passaged Strains Following Intracerebral Inoculation

To compare the neurovirulence of the parental AQ‐19 strain and its serially passaged derivatives, 3‐week‐old ICR mice were randomly assigned to five groups: a DMEM control group, an AQ‐19 group, and three passage‐virus groups infected with F5, F8, or F13, respectively. Each group contained 10 mice. Mice in the virus‐infected groups were intracerebrally inoculated under anesthesia with 30 μL of the corresponding virus preparation at a titer of 10^6^ TCID_50_/0.1 mL, corresponding to ~3 × 10^5^ TCID_50_ per mouse. Control mice received 30 μL of serum‐free DMEM through the same route.

All mice were monitored daily for 14 days for body‐weight changes, clinical signs, and survival. Clinical evaluation and humane endpoint criteria were the same as those described in Section 2.3. Mice that reached the predefined humane endpoints were humanely euthanized and recorded as deaths for survival analysis. Body‐weight measurements were discontinued after death or euthanasia. Mice that remained alive at 14 dpi were humanely euthanized at the end of the experiment.

### 2.6. Acquisition of Neuroinvasive Capacity by Serially Passaged Strains Following I.N. Inoculation

To determine whether serial passage altered the neuroinvasive capacity of DTMUV, 3‐week‐old ICR mice were randomly assigned to five groups: a DMEM control group and four virus‐infected groups inoculated with AQ‐19, F5, F8, or F13, respectively. Each group contained 10 mice. Mice in the virus‐infected groups were intranasally inoculated without anesthesia with 100 μL of the corresponding virus preparation at a titer of 10^6^ TCID_50_/0.1 mL, corresponding to 1 × 10^6^ TCID_50_ per mouse. The inoculum was administered equally between the two nostrils. Control mice received 100 μL of serum‐free DMEM using the same procedure.

All mice were monitored daily for 14 days for body‐weight changes, clinical signs, and survival. Clinical evaluation and humane endpoint criteria were the same as those described in Section 2.3. Mice that reached the predefined humane endpoints were humanely euthanized and recorded as deaths for survival analysis. Body‐weight measurements were discontinued after death or euthanasia. Mice that remained alive at 14 dpi were humanely euthanized at the end of the experiment.

Brain tissues were collected from three mice per group for RT‐PCR detection and gross pathological examination. In animals that developed disease, tissues were collected at death or when humane endpoints were reached. In animals that remained clinically normal, tissues were collected at 14 dpi.

To determine whether the adapted phenotype extended to systemic peripheral routes, additional groups of mice were inoculated intraperitoneally or subcutaneously with either AQ‐19 or F13. Each group contained 10 mice, and each mouse received 100 μL of virus at a titer of 10^6^ TCID_50_/0.1 mL. Route‐matched control mice received an equivalent volume of serum‐free DMEM. Mice were monitored daily for 14 days for body‐weight changes, clinical signs, and survival. At 14 dpi, brain tissues were collected from three mice per group for RT‐PCR detection of DTMUV RNA.

### 2.7. CNS Tissue Distribution and Temporal Dissemination of F13 Following I.N. Infection

To characterize the tissue distribution and temporal dissemination of the mouse‐adapted F13 strain following I.n. infection, 3‐week‐old ICR mice were assigned to an F13‐infected group and a DMEM control group. The infected group contained 30 mice, whereas the control group contained five mice. Mice in the infected group were intranasally inoculated without anesthesia with 100 μL of F13 at a titer of 10^6^ TCID_50_/0.1 mL, corresponding to 1 × 10^6^ TCID_50_ per mouse. The inoculum was divided equally between the two nostrils. Control mice received 100 μL of serum‐free DMEM by the same route.

Of the 30 F13‐infected mice, 27 were assigned to the temporal viral‐distribution analysis. Three mice were sampled at each time point from 1 to 9 dpi. During the early phase of infection, when no overt clinical disease was observed, three mice were humanely euthanized at each scheduled time point. During the later phase, mice that died or reached predefined humane endpoints on a given day were included in the corresponding time‐point sampling. When fewer than three mice were available through these events, additional infected mice were humanely euthanized to obtain a total of three animals for that time point.

The olfactory epithelium, olfactory bulb, cerebrum, cerebellum, brainstem, spinal cord, peripheral blood, and visceral organs were collected separately from each animal. Samples from individual mice were processed independently and used exclusively for virological analysis. Viral RNA levels in the olfactory epithelium, olfactory bulb, brain, and spinal cord were quantified by RT‐qPCR to assess the temporal progression of viral dissemination. Regional viral distribution within the CNS, including the olfactory bulb, cerebrum, cerebellum, brainstem, and spinal cord, was also evaluated by RT‐qPCR.

The five DMEM‐treated control mice were maintained until 9 dpi and were then humanely euthanized for collection of the corresponding tissues as endpoint negative controls.

### 2.8. Neuropathological Analysis of F13‐Infected Mice

The remaining three F13‐infected mice were used specifically for neuropathological analyses and were not included in the 1–9 dpi temporal viral‐load study described in Section 2.7. Brain and spinal cord tissues were collected when these mice developed evident neurological disease or reached predefined humane endpoints. Corresponding tissues collected from three DMEM‐treated mice at 9 dpi were used as negative controls.

The collected CNS tissues were fixed and processed for histopathological, immunohistochemical (IHC), and TUNEL analyses. Sections of the olfactory bulb, cerebrum, cerebellum, brainstem, and spinal cord were stained with hematoxylin and eosin (H&E) to evaluate histopathological alterations. Viral antigen distribution within the CNS was examined by immunohistochemistry using an antibody against the DTMUV E protein. Neuronal cell death was further assessed in brain sections using the terminal deoxynucleotidyl transferase dUTP nick‐end labeling assay. All H&E, IHC, and TUNEL analyses were performed using tissues from three mice per group, and representative images are shown. Detailed procedures for tissue processing, immunohistochemistry, and TUNEL staining are described in the corresponding methodological sections below.

### 2.9. Functional Validation of Olfactory Pathway‐Mediated Neuroinvasion

To verify the disruptive effect of ZnSO_4_ on the olfactory epithelium, 3‐week‐old ICR mice were randomly assigned to a ZnSO_4_‐treated group and a PBS‐treated control group, with three mice per group. Mice in the ZnSO_4_‐treated group were intranasally administered 60 μL of 0.17 mol/L ZnSO_4_ without anesthesia, whereas control mice received an equivalent volume of PBS by the same route. The solution was divided equally between the two nostrils. At 24 h after treatment, all mice were humanely euthanized, and the heads were collected and submitted to a commercial histopathology service for tissue processing and H&E staining to evaluate structural damage to the olfactory epithelium.

To determine whether disruption of the olfactory epithelium affected F13 neuroinvasion, 3‐week‐old ICR mice were randomly assigned to four groups: PBS + DMEM, ZnSO_4_ + DMEM, PBS + F13, and ZnSO_4_ + F13, with 25 mice per group. Mice were intranasally administered 60 μL of either 0.17 mol/L ZnSO_4_ or PBS without anesthesia, with 30 μL delivered into each nostril. At 24 h after pretreatment, mice in the virus‐infected groups were intranasally inoculated without anesthesia with 100 μL of F13 at a titer of 10^6^ TCID_50_/0.1 mL, corresponding to 1 × 10^6^ TCID_50_ per mouse. The viral inoculum was divided equally between the two nostrils. Mice in the corresponding control groups received 100 μL of serum‐free DMEM by the same route.

Within each group, 10 mice were monitored daily for 14 days for body‐weight changes, clinical signs, and survival. Clinical evaluation and humane endpoint criteria were the same as those described in Section 2.3. Mice that reached predefined humane endpoints were humanely euthanized and recorded as deaths for survival analysis. Body‐weight measurements were discontinued after death or euthanasia. Animals that remained alive at 14 dpi were humanely euthanized at the end of the experiment.

An additional 12 mice per group were used for temporal analysis of viral replication. Three mice from each group were humanely euthanized at 1, 4, 7, and 9 dpi. The olfactory epithelium and brain tissues were collected separately from each animal, processed independently, and subjected to RT‐qPCR analysis for quantification of DTMUV RNA. The remaining three mice in each group were used for gross pathological examination. In infected groups, tissues were collected when animals developed evident disease or reached predefined humane endpoints; animals that remained clinically normal were examined at the end of the experiment.

### 2.10. RNA Extraction and DTMUV Detection

RNA extraction and DTMUV RT‐PCR methods were previously described [6]. Quantitative RT‐PCR determined the DTMUV RNA copy number. The amplification reaction was conducted in a final volume of 20 μL using the following components: 2 μL cDNA, 0.4 μL of each 20 μM primer, 10 μL ChamQ Universal SYBR qPCR Master Mix (Vazyme, Nanjing, China), and an appropriate volume of ddH_2_O to reach 20 μL. The qPCR profile condition was as follows: 95°C for 3 min, 40 cycles of 95°C for 10 s, 60°C for 30 s. The following primer sequences were used for diagnosis: 5^′^‐AGGAAGTGGAGCAATCAGGAA‐3^′^ (forward) and 5^′^‐TAACAAGTGGCAGAGCAAAGG‐3^′^ (reverse).

### 2.11. Histopathology and Immunohistochemistry

The brain and other tissue samples were fixed in 4% formaldehyde (Sinopharm Chemical Reagent Co., Ltd., Shanghai, China) in PBS, embedded in paraffin, and sectioned at a thickness of 5 μm using a Leica SM2010R microtome (Leica, Shanghai, China). For histopathological examination, tissue sections were stained with H&E (Beyotime Biotechnology, Shanghai, China) and examined under an Eclipse E100 light microscope at 400× magnification (Nikon, Japan).

For IHC staining, paraffin‐embedded sections were deparaffinized, rehydrated, subjected to antigen retrieval, and blocked to reduce endogenous peroxidase activity and nonspecific binding. The sections were then incubated overnight at 4°C with a monoclonal antibody against the DTMUV E protein prepared in our laboratory at a dilution of 1:1000. After washing with PBS, the sections were incubated with a horseradish peroxidase‐conjugated secondary antibody at a dilution of 1:2000 (ab97040, Abcam). Immunoreactivity was visualized using 3,3^′^‐diaminobenzidine as the chromogenic substrate, followed by counterstaining with hematoxylin. The stained sections were examined under an Eclipse E100 light microscope (Nikon, Japan).

### 2.12. Terminal Deoxynucleotidyl Transferase dUTP Nick‐End Labeling (TUNEL)

Brain tissue sections were dewaxed in xylene (Sigma–Aldrich, USA) for 5–10 min, followed by another dewaxing step with fresh xylene for 5–10 min. Subsequently, the sections were dehydrated in 100% ethanol for 5 min, 90% ethanol for 2 min, 70% for 2 min, and distilled water for 2 min. Proteinase K (20 μg/mL) without DNase (Thermo Fisher Scientific, USA) was added dropwise, and the sections were incubated at 37°C for 15–30 min. The sections were then washed with PBS three times for 5 min each. Next, 50 μL of TUNEL detection solution (Thermo Fisher Scientific, USA) was added to each sample, followed by incubation at 37°C in the dark for 60 min. The sections were washed with PBS three times. Finally, the sections were sealed with an antifluorescence quenching mountant (Yuanye Biotechnology Co., Ltd, Shanghai, China) and observed under a fluorescence microscope.

### 2.13. Genetic Variation Analysis of DTMUV AQ‐19 F13

For each selected passage strain (F5, F8, and F13), the virus used for sequence analysis was recovered from pooled brain tissues collected from five affected mice during the corresponding passage. The pooled brain homogenate was propagated once in BHK‐21 cells, and the resulting virus stock was used for sequence analysis. Thus, one pooled virus sample was analyzed for each passage strain, and the identified substitutions represent the consensus sequence of the corresponding pooled virus population rather than mutations independently confirmed in individual mice.

The full‐length genome of DTMUV AQ‐19 F13 was amplified using an overlapping RT‐PCR method. The 50 µL reaction mixtures were set up with 25 µL 2×Phanta Max Master Mix (Vazyme, Nanjing, China), 1 µL of each 10‐µM primer, 2 µL cDNA, and 21 µL ddH_2_O. The PCR products were purified and cloned into pCE2 TA/BluntZero vector (Vazyme, Nanjing, China), and clones were obtained for sequencing (Tsingke, Nanjing, China) to determine the correct genome information. We used DNAMAN v.6.0 software (Lynnon Biosoft, San Ramon, CA, USA) and Lasergene.v7.1 software (DNAStar, Madison, WI, USA) to splice different fragments to obtain the correct full‐length genome of AQ‐19 F13. The evolutionary analysis of the nucleotide and amino acid sequences of the coding protein genes of AQ‐19 F13 and other DTMUV strains was conducted using MEGA v.11.0 software and Lasergene.v7.1 software (DNAStar) with the Clustal W method.

### 2.14. Statistical Analysis

Quantitative data are presented as the mean ± standard deviation (SD), with each mouse considered an independent biological replicate. Survival curves were generated using the Kaplan–Meier method and compared using the log‐rank (Mantel–Cox) test. Body‐weight data and viral RNA levels measured in different treatment groups, tissues, or sampling time points were analyzed using two‐way analysis of variance (ANOVA). Histopathological, IHC, gross pathological, RT‐PCR, and TUNEL results were evaluated qualitatively, and representative images from three mice per group are shown. Statistical analyses were performed using GraphPad Prism 8.0 (GraphPad Software, San Diego, CA, USA). A two‐sided *p* value < 0.05 was considered statistically significant ( ^∗^ *p* < 0.05;  ^∗∗^ *p* < 0.01).

## 3. Results

### 3.1. DTMUV AQ‐19 Exhibits Neurovirulence but Limited Neuroinvasive Capacity in Mice

**Figure 1:**
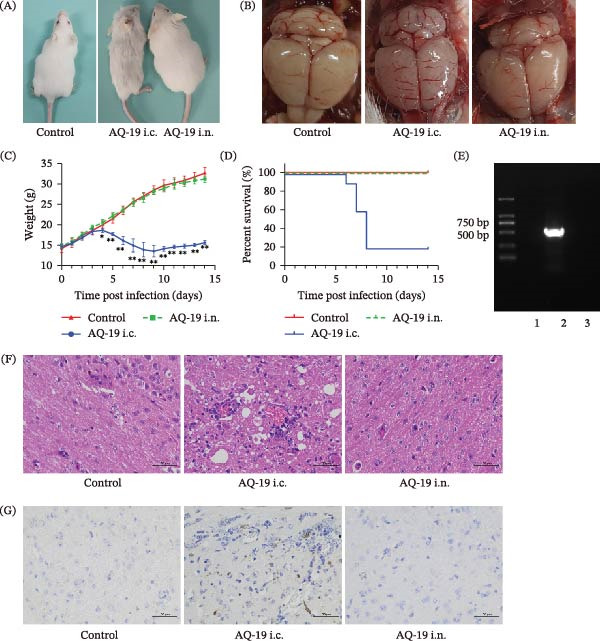
Neurovirulence and limited neuroinvasive capacity of DTMUV AQ‐19 in mice. (A) Clinical signs observed in mice following intracranial (i.c.) or intranasal (i.n.) inoculation with DTMUV AQ‐19, including neurological symptoms such as depression and hind limb paralysis in the i.c. group. (B) Gross pathological changes in brain tissues, showing progressive hemorrhagic lesions in i.c.‐infected mice, while no visible abnormalities were observed in i.n. or control groups. (C) Body weight changes of mice following infection via different routes. (D) Survival curves of mice infected with DTMUV AQ‐19 via i.c. or i.n. routes. (E) Detection of viral RNA in brain tissues by RT‐PCR. Viral RNA was detected in the i.c. group but not in the i.n. or control groups. (F) Histopathological analysis of brain tissues (H&E staining), showing features of encephalitis, including vascular congestion, perivascular cuffing, inflammatory cell infiltration, and neuronal damage in the i.c. group. (G) Immunohistochemical (IHC) detection of viral antigen in brain tissues, showing widespread antigen distribution in the i.c. group, with no positive signals in the i.n. or control groups. Panel A shows representative clinical images from the cohort of 10 mice per group. Body‐weight and survival analyses included 10 mice per group at the beginning of the experiment. Gross pathological examination, RT‐PCR, H&E staining, and IHC were performed using tissues from three mice per group, and representative results are shown. Body‐weight data are presented as mean ± SD and were analyzed using two‐way repeated‐measures ANOVA. Survival curves were compared using the log‐rank (Mantel–Cox) test. A *p* value < 0.05 was considered statistically significant ( ^∗^ *p* < 0.05;  ^∗∗^ *p* < 0.01).

To determine whether DTMUV AQ‐19 can infect mice via different routes, mice were inoculated either intracranially or intranasally, and disease progression was systematically evaluated. Following intracranial inoculation, mice developed evident neurological symptoms, including depression, rough coat, and hind limb paralysis (Figure 1A). Gross examination revealed progressive hemorrhagic lesions in the brain tissues of infected mice, whereas no visible abnormalities were observed in the i.n. or control groups (Figure 1B). Consistently, intracranially infected mice exhibited continuous weight loss and significant mortality, reaching up to 80% by 14 dpi (Figure 1C,D). In contrast, i.n. inoculation of AQ‐19 did not result in observable clinical signs, weight loss, or mortality, and mice remained comparable to the control group throughout the observation period (Figure 1A–D).

Molecular and histopathological analyses further confirmed these findings. Viral RNA was readily detected in the brain tissues of intracranially infected mice but was undetectable in the i.n. and control groups (Figure 1E). Histopathological examination revealed typical features of viral encephalitis, including vascular congestion, perivascular cuffing, inflammatory cell infiltration, and neuronal damage, exclusively in the intracranial infection group (Figure 1F). In addition, immunohistochemistry showed widespread viral antigen distribution in the CNS following intracranial inoculation, whereas no positive signals were observed in the i.n. or control groups (Figure 1G).

Together, these results demonstrate that DTMUV AQ‐19 is capable of replicating in the CNS and inducing severe neuropathological damage following direct intracranial inoculation. However, its inability to establish infection via the i.n. route indicates that AQ‐19 lacks efficient neuroinvasive capacity following nonintracranial exposure. These findings suggest that host barriers encountered outside the CNS prevent AQ‐19 from gaining access to the brain, despite its ability to replicate and cause disease once directly introduced into neural tissues.

### 3.2. Serial Intracranial Passage Enhances the Neurovirulence of DTMUV in Mice

**Figure 2:**
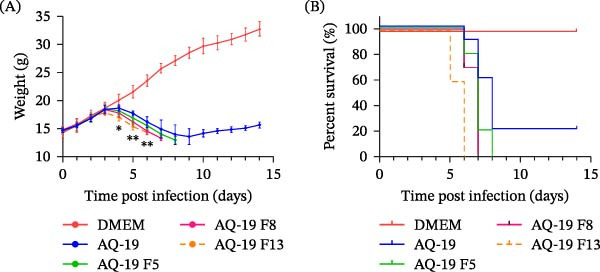
Serial intracranial passage enhances the neurovirulence of DTMUV in mice. (A) Body weight changes of mice following intracranial infection with the parental AQ‐19 strain and its serially passaged derivatives (F5, F8, and F13). Mice infected with passaged viruses exhibited more rapid and pronounced weight loss compared with those infected with the parental strain. (B) Survival curves of mice infected with AQ‐19 and passaged viruses. Serially passaged strains showed accelerated mortality, with earlier onset of death observed across successive passages. Ten mice were included in each group at the beginning of the experiment. Body‐weight data are presented as mean ± SD and were analyzed using two‐way repeated‐measures ANOVA. Survival curves were compared using the log‐rank (Mantel–Cox) test. A *p* value < 0.05 was considered statistically significant ( ^∗^ *p* < 0.05;  ^∗∗^ *p* < 0.01).

To evaluate whether serial intracranial passage promotes viral adaptation in mice, the parental AQ‐19 strain and its passaged derivatives (F5, F8, and F13) were compared in a mouse infection model. Compared with the parental AQ‐19 strain, passaged viruses induced a more rapid decline in body weight, with the magnitude and onset of weight loss progressively increasing across successive passages (Figure 2A). Consistently, survival analysis revealed a marked enhancement in virulence following serial passage. Mice infected with AQ‐19 exhibited delayed disease progression, with neurological symptoms appearing at ~7 dpi and a cumulative mortality of 80% by 14 dpi. In contrast, mice infected with passaged viruses developed symptoms earlier and succumbed more rapidly, with the F13 group displaying the most pronounced phenotype, including onset of neurological signs as early as 5 dpi and 100% mortality by 6 dpi (Figure 2B). Notably, intermediate passage strains (F5 and F8) exhibited phenotypes between those of AQ‐19 and F13, supporting a gradual increase in neurovirulence during serial intracranial passage.

Collectively, these results demonstrate that continuous passage of DTMUV in the mouse brain leads to progressive enhancement of neurovirulence, indicating rapid viral adaptation to the mammalian host.

### 3.3. Mouse‐Adapted DTMUV Acquires Neuroinvasive Ability via Intranasal Inoculation

To determine whether mouse adaptation altered the ability of DTMUV to establish infection through different nonintracranial routes, mice were inoculated with the parental AQ‐19 strain or its passaged derivatives (F5, F8, and F13) via i.n., i.p., or s.c. administration. Consistent with the findings in Section 3.1, the parental AQ‐19 strain failed to establish infection via all tested nonintracranial routes, as no clinical signs, weight loss, or mortality were observed, and viral RNA remained undetectable in brain tissues. Notably, even after adaptation, F13 did not cause infection when administered via i.p. or s.c. routes, with mice remaining clinically normal throughout the 14‐day observation period and no viral RNA detected in the brain (data not shown). These findings indicate that mouse adaptation did not confer a generalized ability to establish infection through systemic peripheral routes.

**Figure 3:**
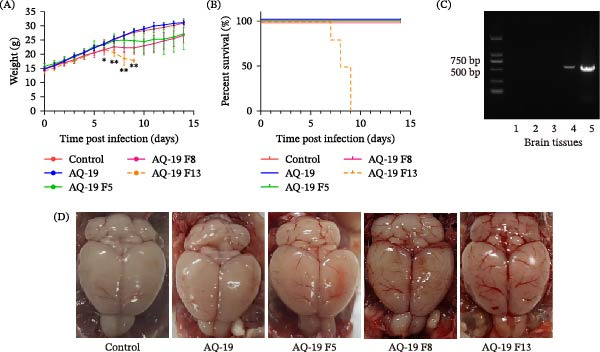
Mouse‐adapted DTMUV acquires neuroinvasive ability via intranasal inoculation. (A) Body weight changes of mice following intranasal infection with the parental AQ‐19 strain and its passaged derivatives (F5, F8, and F13). Mice infected with F13 exhibited marked weight loss, whereas AQ‐19–infected mice remained stable and F5/F8‐infected mice showed only transient weight changes. (B) Survival curves of mice following intranasal infection. F13‐infected mice showed 100% mortality by 9 dpi, while AQ‐19 and lower passage groups (F5 and F8) exhibited no mortality. (C) Detection of viral RNA in brain tissues by RT‐PCR following intranasal inoculation. Robust viral RNA signals were detected in F13‐infected mice, whereas only low levels were observed in F8 and no viral RNA was detected in AQ‐19–infected mice. (D) Gross pathological examination of brain tissues, showing pronounced hemorrhagic lesions in F13‐infected mice, while no visible abnormalities were observed in AQ‐19–infected or control mice. Body‐weight and survival analyses included 10 mice per group at the beginning of the experiment. RT‐PCR and gross pathological examinations were performed using brain tissues from three mice per group, and representative results are shown. Body‐weight data are presented as mean ± SD and were analyzed using two‐way repeated‐measures ANOVA. Survival curves were compared using the log‐rank (Mantel–Cox) test. A *p* value < 0.05 was considered statistically significant ( ^∗^ *p* < 0.05;  ^∗∗^ *p* < 0.01).

In contrast, i.n. inoculation revealed a clear generation‐dependent difference in viral phenotypes. Mice infected with AQ‐19 showed no clinical abnormalities and maintained normal body weight and survival, with no detectable viral RNA in the brain. Mice infected with F5 and F8 exhibited transient weight loss at ~7 dpi, followed by recovery, and only low levels of viral RNA were detected in the brain of F8, without apparent neurological symptoms (Figure 3A–C).

Strikingly, mice infected with the F13 strain developed significant weight loss starting at 7 dpi, followed by rapid disease progression (Figure 3A). Consistently, survival analysis demonstrated that all F13‐infected mice succumbed to infection within a short time frame, with 100% mortality observed by 9 dpi (Figure 3B). Neurological symptoms, including hind limb paralysis and emaciation, were observed prior to death. RT‐PCR analysis further demonstrated robust viral replication in the brain tissues of F13‐infected mice, while viral RNA remained undetectable in serum and peripheral organs, suggesting that viral dissemination to the CNS is not mediated through systemic circulation but likely occurs via neural pathways (Figure 3C). Postmortem examination revealed pronounced hemorrhagic lesions in the brain tissues of F13‐infected mice, whereas no abnormalities were observed in the control group (Figure 3D). Importantly, further i.n. passage of F13 (up to F16) consistently resulted in lethal infection, indicating stable acquisition of neuroinvasive capacity (data not shown).

Collectively, these results demonstrate that serial intracranial passage enables DTMUV to acquire neuroinvasive capacity via a route‐restricted peripheral pathway. Among the tested routes, i.n. inoculation represents the only effective pathway for CNS invasion, strongly suggesting that viral entry occurs through the olfactory system.

### 3.4. Mouse‐Adapted DTMUV Exhibits Strong CNS Tropism and Dynamic Viral Spread

**Figure 4:**
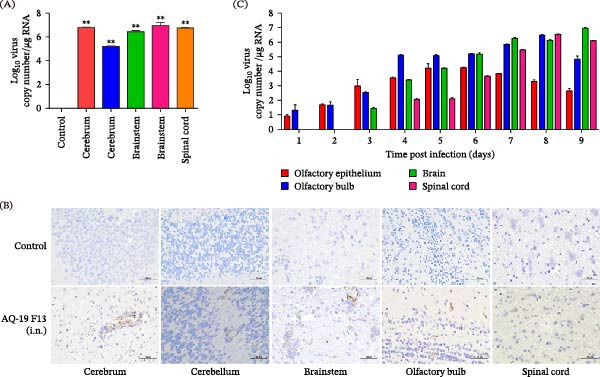
Mouse‐adapted DTMUV exhibits strong CNS tropism and dynamic viral spread following intranasal infection. (A) Quantification of viral RNA levels in different regions of the central nervous system (CNS) by RT‐qPCR, including the olfactory bulb, cerebrum, cerebellum, brainstem, and spinal cord. High viral loads were detected across all examined CNS regions. (B) Immunohistochemical (IHC) detection of viral antigen in CNS tissues, showing widespread antigen distribution in the olfactory bulb, cerebrum, cerebellum, brainstem, and spinal cord. (C) Temporal analysis of viral RNA levels in the olfactory epithelium, brain, and spinal cord following intranasal infection. Viral RNA was first detected in the olfactory epithelium and olfactory bulb at early stages, followed by a rapid increase in the brain and subsequent detection in the spinal cord at later stages. For panel A, viral RNA levels were quantified using tissues from three mice per group, with each mouse analyzed independently as a biological replicate. Viral RNA levels in F13‐infected mice were compared with those in the corresponding tissues from uninfected control mice using two‐way analysis of variance (ANOVA). IHC analysis in panel B was performed using tissues from three mice per group, and representative images are shown. Quantitative data are presented as mean ± SD. A *p* value < 0.05 was considered statistically significant ( ^∗^ *p* < 0.05;  ^∗∗^ *p* < 0.01).

Following successful neuroinvasion via i.n. inoculation, we next investigated the distribution and propagation dynamics of mouse‐adapted DTMUV (F13) within the CNS. RT‐qPCR analysis revealed that viral RNA was abundantly detected across multiple CNS regions, including the olfactory bulb, cerebrum, cerebellum, brainstem, and spinal cord (Figure 4A), whereas viral RNA levels in peripheral tissues remained negligible or undetectable (data not shown). These findings demonstrate a pronounced restriction of viral replication to neural tissues, indicating strong CNS tropism of the adapted virus. Consistently, IHC staining showed widespread viral antigen distribution across corresponding CNS regions (Figure 4B), confirming active infection of diverse neural cell populations and reinforcing the preferential targeting of the CNS by mouse‐adapted DTMUV.

To further define the pattern of viral dissemination, we analyzed the temporal progression of infection following i.n. inoculation. Viral RNA was initially detected in the olfactory epithelium and olfactory bulb at early stages of infection. Subsequently, viral loads increased rapidly throughout the brain, reaching peak levels during the middle stage of infection, whereas viral replication in the spinal cord became evident predominantly at later stages (Figure 4C). This stepwise pattern indicates that infection is established at the nasal entry site and then propagates sequentially through anatomically connected neural structures. Viral spread appears to proceed from the olfactory epithelium to the olfactory bulb, followed by dissemination throughout the brain and eventual involvement of the spinal cord. The delayed detection of viral RNA in the spinal cord suggests a directional spread from the brain to the spinal cord rather than the reverse.

Collectively, these findings demonstrate that mouse‐adapted DTMUV exhibits strong CNS tropism and undergoes a structured and progressive spread within the CNS following i.n. infection, supporting a model of neural pathway‐mediated viral dissemination.

### 3.5. Mouse‐Adapted DTMUV Induces Severe Neuropathological Damage in Mice

**Figure 5:**
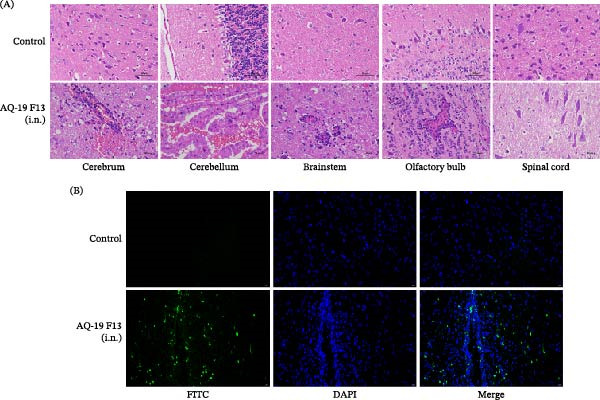
Mouse‐adapted DTMUV induces severe neuropathological damage in the central nervous system. (A) Histopathological analysis of CNS tissues by hematoxylin and eosin (H&E) staining. Representative sections from the cerebrum, cerebellum, brainstem, olfactory bulb, and spinal cord show widespread pathological changes, including vascular congestion, perivascular cuffing, inflammatory cell infiltration, neuronal degeneration and necrosis, and glial cell activation in infected mice. (B) Detection of apoptotic cells in brain tissues by TUNEL staining. Extensive apoptotic signals were observed in infected mice, whereas minimal signals were detected in the control group. H&E and TUNEL analyses were performed using tissues from three mice per group, and representative images are shown.

To assess the neuropathological consequences of DTMUV infection following i.n. inoculation, histopathological and apoptotic analyses were performed on CNS tissues from infected mice. H&E staining revealed widespread pathological alterations across multiple regions of the CNS, including the cerebrum, cerebellum, brainstem, olfactory bulb, and spinal cord (Figure 5A). These changes were consistent with nonsuppurative encephalomyelitis, characterized by vascular congestion, perivascular cuffing, inflammatory cell infiltration, neuronal degeneration and necrosis, and glial cell activation.

To further evaluate neuronal damage at the cellular level, TUNEL staining was performed on brain tissues. A substantial increase in apoptotic cells was observed in infected mice compared with controls, indicating extensive neuronal cell death within the CNS (Figure 5B). The widespread distribution of apoptotic signals suggests that neuronal loss is a prominent feature of DTMUV‐induced neuropathology.

Collectively, these findings demonstrate that mouse‐adapted DTMUV infection induces severe neuropathological damage characterized by widespread inflammation and neuronal apoptosis, which likely contributes to the observed neurological dysfunction and fatal disease outcome.

### 3.6. The Olfactory Epithelium Serves as a Major Route for Nasal‐to‐Brain Transmission of DTMUV

**Figure 6:**
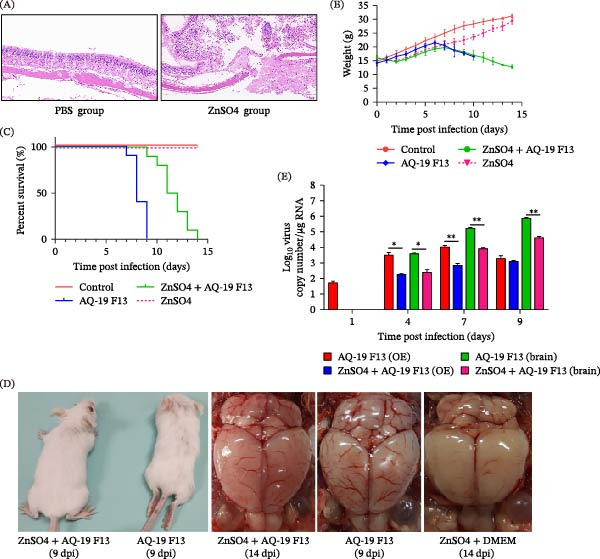
Disruption of the olfactory epithelium reduces DTMUV neuroinvasion following intranasal infection. (A) Histological analysis of the olfactory epithelium by hematoxylin and eosin (H&E) staining. ZnSO_4_‐treated mice exhibited marked epithelial damage, including disorganization and cellular exfoliation, whereas control mice showed intact epithelial structure. (B) Body weight changes of mice following intranasal infection with AQ‐19 F13, with or without ZnSO_4_ pretreatment. (C) Survival curves of infected mice. ZnSO_4_ pretreatment delayed mortality compared with control mice. (D) Representative disease progression following infection, showing delayed onset of neurological symptoms in ZnSO_4_‐treated mice compared with controls. (E) Quantification of viral RNA levels in the olfactory epithelium and brain tissues by RT‐qPCR. Viral loads were significantly reduced in ZnSO_4_‐treated mice compared with controls. For panel A, histopathological examination was performed using three mice per group, and representative H&E‐stained sections are shown. For panels B and C, 10 mice per group were included at the beginning of the body‐weight and survival analyses. For panel D, clinical observations were performed in three mice per group, and representative images are shown. For panel E, three mice per group were analyzed independently at each time point, with each mouse representing one biological replicate. Quantitative data are presented as mean ± SD. Body‐weight changes were analyzed using two‐way ANOVA. Survival curves were compared using the log‐rank (Mantel–Cox) test. Viral RNA levels in the olfactory epithelium and brain were analyzed separately using two‐way ANOVA. A *p* value < 0.05 was considered statistically significant ( ^∗^ *p* < 0.05;  ^∗∗^ *p* < 0.01).

Given that DTMUV infection in mice was strictly dependent on the i.n. route and could not be established via other peripheral routes, we hypothesized that the virus may access the CNS through a direct anatomical pathway connecting the nasal cavity to the brain. The olfactory epithelium, a well‐recognized entry interface for neurotropic viruses, was therefore investigated as a potential route for DTMUV neuroinvasion. To test this hypothesis, mice were pretreated with ZnSO_4_ to selectively disrupt the olfactory epithelium prior to i.n. infection. Histological analysis confirmed that ZnSO_4_ treatment caused marked structural damage to the olfactory epithelium, including epithelial disorganization and cellular exfoliation, whereas the control group maintained intact epithelial architecture (Figure 6A), indicating effective impairment of the olfactory barrier.

Following i.n. inoculation with AQ‐19 F13, ZnSO_4_‐pretreated mice exhibited a significantly delayed onset of disease compared to PBS‐treated infected controls. While control mice developed severe neurological symptoms and succumbed to infection within 7–9 dpi, ZnSO_4_‐treated mice showed only transient weight loss at early time points, with disease onset and mortality delayed to 10–14 dpi (Figure 6B–D). These findings indicate that impairment of the olfactory epithelium reduces the efficiency of DTMUV neuroinvasion.

Consistent with these findings, RT‐qPCR analysis demonstrated significantly reduced viral loads in both the olfactory epithelium and brain tissues of ZnSO_4_‐treated mice compared to controls (Figure 6E). Viral RNA was first detected in the olfactory epithelium at early stages of infection, supporting its role as an initial site of viral entry.

Collectively, these results demonstrate that the olfactory epithelium plays a critical role in facilitating DTMUV entry into the CNS following i.n. exposure.

### 3.7. Mouse Adaptation of DTMUV Is Associated With Specific Amino Acid Mutations

**Figure 7:**
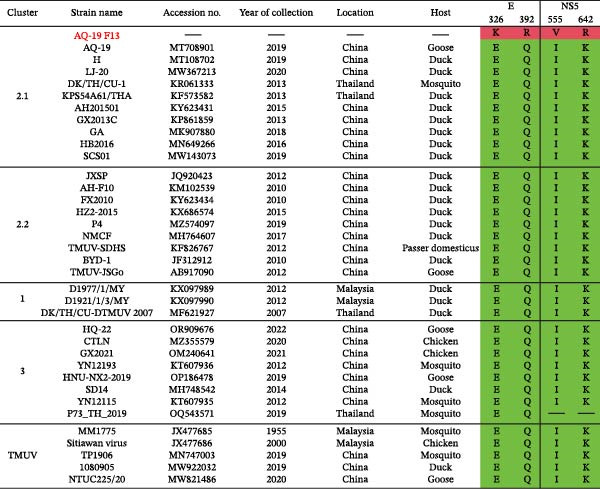
Identification of amino acid substitutions associated with mouse adaptation of DTMUV. Comparison of the full‐length genome sequences of the parental AQ‐19 strain and the mouse‐adapted F13 strain revealed four amino acid substitutions. The positions and identities of these nonsynonymous mutations are indicated.

To investigate the genetic basis underlying the enhanced neurovirulence and neuroinvasive capacity of the mouse‐adapted DTMUV strain, the complete genome sequence of AQ‐19 F13 was determined and compared with that of the parental AQ‐19 strain. The full‐length genome of F13 was successfully assembled and found to be identical in length (10,991 nt) to that of the parental virus. Sequence alignment identified only four amino acid substitutions in the F13 strain compared with AQ‐19 (Figure 7). Specifically, two substitutions were located in the envelope (E) protein (E326K and Q392R), whereas the other two were located in the NS5 protein (I555V and K642R). Notably, all mutations were nonsynonymous, suggesting potential functional significance in viral adaptation.

To investigate the sequential accumulation of adaptive mutations during mouse passage, the corresponding genomic regions of the intermediate passage strains F5 and F8 were also analyzed. Sequence comparison revealed that both F5 and F8 contained only a single amino acid substitution (Q392R) in the E protein relative to the parental AQ‐19 strain, whereas the additional substitutions (E326K, I555V, and K642R) were detected only in the F13 strain (Figure 7). Thus, the acquisition of adaptive mutations was not simultaneous but occurred progressively during serial mouse passage. Notably, the Q392R substitution was consistently detected from F5 onward, whereas the remaining three substitutions appeared only in the fully adapted F13 strain, coinciding with the acquisition of robust neuroinvasive capacity.

The limited number of amino acid substitutions identified in F13 suggests that only a small number of genetic changes may be associated with the emergence of olfactory route‐mediated neuroinvasive capacity during mammalian adaptation. This finding raises the possibility that relatively few adaptive mutations may contribute to overcoming species‐specific barriers to mammalian neuroinvasion. However, because reverse genetics validation was not performed in the present study, the individual contribution of each substitution and their potential combinatorial effects remain to be determined. Further studies using reverse genetics will be required to determine whether these substitutions represent key determinants of DTMUV adaptation to mammalian hosts.

## 4. Discussion

DTMUV has undergone continuous evolution in avian hosts in recent years, accompanied by an expansion of host range and increased virulence [10, 11, 18, 23]. These changes raise an important question as to whether emerging DTMUV variants have the potential to cross species barriers and infect mammals. In the present study, we addressed this question by systematically evaluating the infection capacity of a recently circulating variant, AQ‐19, in a mouse model. Our findings demonstrate that, despite enhanced virulence, AQ‐19 remains unable to establish infection via peripheral routes, indicating that a substantial species barrier still restricts DTMUV transmission to mammals. Notably, we further show that this barrier can be overcome following limited adaptive changes, highlighting a critical transition from restricted host tropism to mammalian neuroinvasion.

The observation that AQ‐19 exhibits strong neurovirulence following intracranial inoculation but fails to establish infection via peripheral routes underscores a fundamental distinction between viral pathogenicity and cross‐species transmission capacity. While intracranial inoculation directly exposes the CNS and bypasses early host barriers, successful peripheral infection requires the virus to efficiently enter host cells, replicate in peripheral tissues, and disseminate to the CNS [24]. The inability of AQ‐19 to complete these steps in mice suggests that, despite ongoing evolution in avian hosts, DTMUV has not yet acquired the necessary adaptations to initiate infection in mammals. This finding is particularly important, as it indicates that enhanced virulence in the natural host does not necessarily translate into an increased risk of cross‐species transmission, and that a functional barrier still exists at the level of peripheral infection.

Building on this observation, we further investigated whether additional adaptive changes could enable DTMUV to overcome this barrier. Through serial passage in mice, we identified a mouse‐adapted strain that acquired the ability to infect via the i.n. route and cause lethal disease. Notably, this phenotypic shift was associated with only four nonsynonymous amino acid substitutions, suggesting that TMUV possesses the evolutionary potential to acquire mammalian neuroinvasive capacity through relatively limited genetic changes. Similar patterns have been observed in other flaviviruses, where limited mutations can markedly alter host range, virulence, or tissue tropism [25–30]. Our findings therefore indicate that DTMUV may possess a low genetic barrier for host adaptation. Notably, despite acquiring efficient neuroinvasion following i.n. inoculation, the mouse‐adapted F13 strain remained unable to establish productive infection after i.p. or s.c. inoculation. This observation suggests that the adaptive mutations selected during serial intracranial passage preferentially enhanced neural invasion and/or viral fitness within the CNS rather than conferring efficient systemic dissemination in mammals. Consistent with this interpretation, viral replication in F13‐infected mice was predominantly restricted to the CNS, in contrast to the dissemination to visceral organs reported for some DTMUV strains following intracerebral inoculation. This difference may reflect strain‐specific tissue tropism and the selective pressure imposed by repeated passage in the mouse brain, which would be expected to favor replication and spread within neural tissues without necessarily enhancing fitness in peripheral organs. In addition, the markedly accelerated disease course caused by F13 may have shortened the time available for detectable viral dissemination and accumulation in visceral tissues. Therefore, the higher virulence of F13 should be interpreted primarily as enhanced neurovirulence rather than broader systemic pathogenicity. Thus, the adaptive phenotype observed in F13 represents a partial, route‐dependent form of mammalian adaptation rather than complete adaptation for peripheral infection. Nevertheless, these findings raise concerns that continued viral evolution could facilitate the emergence of strains with progressively enhanced capacity for cross‐species transmission.

To better understand the potential biological functions of the genes harboring the identified mutations and their implications for mammalian adaptation, the four amino acid substitutions identified in the mouse‐adapted F13 strain warrant further consideration. Two substitutions (E326K and Q392R) were located in the envelope (E) protein, whereas the remaining two (I555V and K642R) were located in NS5. The flaviviral E protein is a major determinant of receptor binding, membrane fusion, tissue tropism, and host range [31]. In JEV and other neurotropic flaviviruses, variation in the E protein has been associated with altered neurovirulence, tissue tropism, and host adaptation, highlighting the important role of E protein evolution during mammalian adaptation [29, 30, 32]. In contrast, NS5 is the largest and most conserved flaviviral protein, functioning not only as the viral RNA‐dependent RNA polymerase and methyltransferase but also as a key antagonist of host innate immune responses [33]. Mutations in NS5 have been reported to alter viral RNA replication, interferon antagonism, and host‐dependent viral fitness, underscoring its multifunctional role in flavivirus evolution and host adaptation [34, 35]. It is therefore conceivable that these substitutions influence distinct stages of mammalian adaptation, with E substitutions potentially facilitating early events involved in host‐cell entry, tissue tropism, and neuroinvasion, whereas NS5 substitutions may enhance viral replication or immune evasion during subsequent stages of infection. The coexistence of substitutions in both structural (E) and nonstructural (NS5) proteins further suggests that successful mammalian adaptation may require coordinated optimization of multiple stages of the viral life cycle, rather than alteration of a single viral function. However, an important limitation of the present study is that the functional roles of these substitutions were not directly evaluated. Consequently, it remains unclear whether the observed phenotype results from a single dominant mutation, the combined effects of multiple mutations, or interactions among them. Future studies using reverse genetics will therefore be required to define the individual and combined contributions of these substitutions to mammalian adaptation and neuroinvasion.

TMUV exhibits a complex transmission pattern in avian hosts. In addition to mosquito‐borne transmission, several duck‐origin strains can infect birds through the i.n. route and spread through direct contact or aerosols, although this capacity varies among viral strains and host species [3, 36–38]. Therefore, i.n., i.p., and s.c. inoculation were examined in parallel in the present study to determine whether mouse adaptation altered the ability of DTMUV to establish infection through different exposure routes. F13 remained noninfectious following i.p. and s.c. inoculation but caused lethal CNS infection after i.n. exposure, indicating that the acquired phenotype was strongly route‐dependent. However, this finding should not be interpreted as evidence of respiratory transmissibility, because viral shedding and animal‐to‐animal transmission were not evaluated. The temporal and anatomical distribution of viral RNA further distinguished this phenotype from systemic infection. Viral RNA was detected initially in the olfactory epithelium and olfactory bulb, while remaining undetectable in blood and peripheral organs, and subsequently spread to other regions of the brain and spinal cord. This distribution pattern is consistent with direct nasal‐to‐brain invasion rather than hematogenous dissemination. Moreover, disruption of the olfactory epithelium significantly reduced early viral loads in the olfactory epithelium and brain and delayed disease onset, providing functional evidence that the olfactory pathway substantially contributes to CNS entry. Taken together, these findings indicate that serial intracranial passage did not confer broad infectivity through multiple peripheral routes but instead selected a restricted mammalian‐adaptive phenotype characterized by efficient olfactory route‐mediated neuroinvasion and subsequent replication within the CNS.

**Figure 8:**
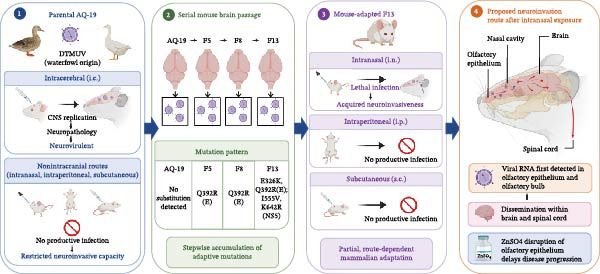
Proposed model of mouse adaptation and olfactory route‐mediated neuroinvasion of DTMUV. The parental AQ‐19 strain was neurovirulent following intracerebral inoculation but failed to establish infection through nonintracranial routes. Serial intracranial passage resulted in stepwise accumulation of adaptive mutations and generated the F13 strain, which exhibited enhanced neurovirulence and acquired lethal intranasal neuroinvasion while remaining noninfectious following intraperitoneal or subcutaneous inoculation. After intranasal exposure, F13 entered the CNS through the olfactory pathway and subsequently disseminated throughout the brain and spinal cord. Disruption of the olfactory epithelium delayed disease progression, supporting its role in nasal‐to‐brain transmission.

Collectively, our study demonstrates that although currently circulating DTMUV variants remain restricted in their ability to infect mammals via peripheral routes, this barrier can be overcome following limited adaptive genetic changes under experimental selection conditions. The identification of a limited number of adaptive genetic changes associated with mammalian neuroinvasion, together with the ability to utilize the olfactory route for CNS invasion, provides a potential experimental framework for understanding how DTMUV may acquire mammalian neuroinvasive capacity through adaptive evolution. The proposed model of mouse adaptation and olfactory route‐mediated neuroinvasion is summarized in Figure 8. Importantly, these findings do not indicate that currently circulating DTMUV strains possess natural mammalian transmissibility but rather reveal a potential evolutionary route by which mammalian adaptation can occur under strong experimental selection. Nevertheless, this study is limited to a mouse model generated by serial intracranial passage, and further investigations are required to determine whether similar adaptive trajectories can occur during natural infection or in other mammalian species. Continuous surveillance and functional characterization of emerging DTMUV variants will therefore be essential to assess and mitigate potential cross‐species transmission risks.

## Funding

This work was supported by the National Natural Science Foundation of China (Grants 32072874 and 32473001), the China Postdoctoral Science Foundation (Grant 2024M753586), the Anhui Provincial Poultry Industry Technology System (Grant AHCYJSTX06), and the University Synergy Innovation Program of Anhui Province (Grant GXXT‐2022‐057).

## Conflicts of Interest

The authors declare no conflicts of interest.

## Data Availability

The data that support the findings of this study are available from the corresponding author upon reasonable request.
